# Mixed methods evaluation of the impact of a short term training program on sterile processing knowledge, practice, and attitude in three hospitals in Benin

**DOI:** 10.1186/s13756-018-0312-6

**Published:** 2018-02-13

**Authors:** Olive Fast, Christina Fast, Dan Fast, Suzanne Veltjens, Zouliha Salami, Michelle White

**Affiliations:** 1 Mount Royal University, Calgary, Canada; 2 Sterile Processing Education Charitable Trust, 5115 Vanstone Cres NW, Calgary, AB T3A 0W1 Canada; 3 Mercy Ships, Cotonou, Benin

**Keywords:** Sterile processing, Education, Africa, Mentoring, Mixed-methods

## Abstract

**Background:**

Proper sterile processing is fundamental to safe surgical practice and optimal patient outcomes. Sterile processing practices in low and middle-income countries often fall short of recommended standards. The impact of education and training on sterile processing practices in low and middle-income countries is unknown. We designed a sterile processing education course, including mentoring, and aimed to evaluate the impact on participants’ personal knowledge, skills, and practices. We also aimed to identify institutional changes in sterile processing practices at participants’ work places.

**Methods:**

A mixed methods design study was conducted using a Hospital Sterile Processing Assessment Tool, knowledge tests, and open-ended interviews.

**Results:**

Education and mentoring improved how workers understood and approached their work and to what they paid attention. Sterile processing workers were also better able to identify resources available to do their work and showed improved understanding of the impact of their work on patient safety.

**Conclusions:**

Health care organizations seeking to improve surgical outcomes can find easy wins requiring minimal cost expenditures by paying attention to sterile processing practices. Investing in education and low-cost resources, such as cleaning detergents and brushes, must be part of any quality improvement initiative aimed at providing safe surgery in low and middle-income countries.

**Electronic supplementary material:**

The online version of this article (10.1186/s13756-018-0312-6) contains supplementary material, which is available to authorized users.

## Background

Proper sterile processing (SP) is fundamental to successful surgeries and improved patient outcomes, yet low and middle income countries (LMICs) often do not meet the World Health Organization’s (WHO) recommended standards [1–4]. SP standards of practice [1] cover the period from cessation of instrument use at the end of one surgery, through to next instrument usage. SP standards cover: transfer of instruments from the operating room (OR) to the decontamination area, the cleaning and decontamination processes, inspection and packaging of instruments, sterilization, instrument storage, and instrument transport back to the OR.

The instrument sterilization process can break down anywhere along the SP pathway. For example, without properly cleaning instruments before autoclaving, microorganisms survive under bioburden that is “baked on”, forming a hard, protective coating during the autoclave process. Later, when this protective coating is dislodged during surgical procedures, microorganisms are freed to infect patients [5].

Available evidence highlights the poor condition of instruments, lack of resources, and lack of standards for surgical device  reprocessing in LMICs [6]. Frequently, reprocessing of surgical devices in LMICs is done by individuals with little to no training, increasing the risk of missing steps to ensure sterility during the SP process [4].

Increased capacity building, staff education, and raising practice standards has been identified as a major way to decrease infection rates in LMICs [6, 7]. While studies call for increased education and training in SP [4, 7], no studies were found that directly identified how education and training of SP staff impacted SP standards of practice in LMICs. To address this gap, we designed a short training program in Benin, with widespread scale up in mind, and evaluated the impact at 4 months.

## Methods

We have used a mixed-methods research design [8] that incorporates both qualitative and quantitative research approaches to address issues in sterile processing. Our use of qualitative data (interviews and hospital assessments) is intended to assess the validity of quantitative data (test results) to strengthen the study [8, 9].

Sterile Processing Education Charitable Trust (SPECT) is a charitable non-governmental organization that provides education and training in SP practices for LMICs. From June 2016 to February of 2017 SPECT worked in collaboration with the Ministry of Health in Benin to provide a SP training program for staff from 3 hospitals: a university teaching hospital, a smaller maternity hospital, and a community hospital run by a national church organization. All three hospitals were in a city serving an estimated 1.2 million people.

In this study, we aimed to evaluate participants’ SP knowledge, skills, and practices following education sessions in SP. We also aimed to identify changes in SP processes and practices at participants’ work places.

### Study design

Data collected included (1) pre- and post-training hospital assessments, (2) pre- and post-training participant tests, and (3) participant interviews.

After initial consultation with the Ministry of Health, baseline hospital assessments of SP practices were conducted using a Hospital SP Assessment tool [4].

Participants gave written voluntary consent to participate and in October 2016 underwent a 2-week course (4 h per day). Lecture topics included microbiology, cleaning and decontamination, inspection and packaging, sterilization, storage, and transport of instruments.

Following the 2-week training participants were offered on-going one-on-one mentoring support. Nine out of 36 participants accepted mentoring support 1 day a week for 2 months.

All participants wrote a SP knowledge test (Additional file 1) before training. At 4 months after training (February 2016), hospital SP assessment and participant knowledge tests were repeated. Additionally, semi-structured interviews were conducted using the interview guide shown in Table 1. Interviews were tape-recorded, transcribed, and translated from French to English for data analysis.

**Table 1:** Interview Questions

How have the education sessions impacted what you do at work?
Did you work with SPECT’s mentor? If so, has working with her changed what you do?
Do you do anything differently at work now than you did before the education and training sessions?
Do you feel differently about your work now than you did before you took the education sessions?

### Data analysis

Hospital and participants were anonymized (Hospitals labeled A, B, and C and participants numerically). Data was analyzed using a concurrent embedded mixed methods approach [10]. Qualitative interview data and Hospital SP Assessment Tool data were analyzed using manual inductive thematic analysis. OF and ZS read and coded all the data; then identified and agreed on defined themes as interpreted from the data. The manual analysis was also supported by Dedoose software.

Quantitative data from participant knowledge tests were analyzed using a paired t-test and SPSS software.

## Results

We report on comparisons between the three separate data sets -interviews, pre and post-hospital assessments, and pre and post-test results - to identify changes in SP knowledge, practices, and attitudes [9].

Thirty-six participants were enrolled in the study (24, 2, and 10 from Hospitals A,B and C respectively) At 4 months three were unavailable for repeat knowledge assessments and interview leaving 33 for final analysis. Nine participants agreed to mentoring support and were re-assessed at 4 months.

### Thematic analysis of interviews

Inductive thematic analysis of interviews identified 5 key themes regarding the participants change in practice and attitudes after training.

#### We changed how things are done

Twenty-nine participants stated they had changed how they did their work. Participant A7 understood the need to protect herself during SP and had changed her practice as shown in her speech: “Now we protect ourselves before we do the cleaning and the sterilization” [A7]. Others noted they now wore gloves because they now realized this was important. Two participants at hospital C and four at hospital A indicated they had started routinely using the 3-bucket system [11] to clean instruments. Several participants identified changes in their cleaning of instruments: cleaning instruments under water to decrease risk of aerosol contamination and using a brush and appropriate detergent. Participants from each hospital highlighted providing a safe storage area for sterilized instruments and implementing record keeping.

#### We changed our way of seeing things

Ten participants indicated the training had changed their perception of SP work. They had a greater understanding of why things should be done a certain way, how sterilization equipment worked, and the value of SP work in patient care. This caused a shift in how they felt about their work. Adjectives such as happy, professionally conscious, proud, comfortable, satisfied, and better were used to describe how they felt as shown by the following speeches: “I feel I am giving out quality treatment now” [C6]; “now we are sure of giving out good health quality services to the population.; "now we know we are not putting their health at risk” [C11].

#### Now we pay attention

Nine participants identified paying closer attention to handling and caring for instruments. Inspection became a new and routine part of their cleaning process. C1 noted: “now when we bring out a box ready for surgery and we notice that it is slightly open, or someone has opened it to take an instrument inside, we re-sterilize it again before use.” A13, stated: “now we know the importance of cleaning and disinfection – we respect the different steps. For example, for the cleaning we now take our time to remove the gross soil and blood clots and we inspect (the instrument). We didn’t do that before.” Another frequently mentioned change was stopping the practice of leaving instruments in bleach solution for hours and instead removing instruments after 10–15 min to prevent damage.

Previously hospital A used a dry heat sterilizer however, having learned that steam sterilization (autoclave) is more effective, they had changed their practice and “abandoned the dry heat for the steam sterilizer” [A19]. A prior barrier to autoclave use was the perception that autoclaves were more difficult to use, but after the training they felt more confident and now “made sure everyone used the autoclave” [A20]. Further evidence the knowledge and understanding motivated change in practice is shown by the comment from C1, “before when we worked we did as we liked because we had no idea of how to handle the instruments properly, but now with the training we take our work seriously and do the right thing at the right time.”

#### Decreased surgical site infections

Nine participants from 2 out of 3 hospitals reported a decrease in surgical site infections following training. C9 stated: “the risks of post-surgery infection are really reduced. We have fewer patients that come after surgery with infections.” C6 supported C9s comment: “we used to have cases of post-surgical infections but now it is reduced.” A13 stated:

"we have been wondering where patients got their infections from. We tried cleaning the whole room but there was no difference. But from the training we received we now know that infection can come from the instruments because of the cleaning steps that we neglected. We thank God now and SPECT that we have less infections."

One participant reported visiting the ward each week to see if they had surgical site infections, and stated that the ward reported fewer infections than before the training. Another indicated the surgeons complained less about infections. Inadequate surgical site infection reporting procedures in the hospitals in the study prevented quantitative verification of these comments.

#### Resource concerns

Eight participants mentioned lack of resources as barriers to implementing recommended practices. Specifics were a lack of appropriate detergent (enzymatic cleaner) causing continued use of bleach as a disinfectant. One clinical area did not have an autoclave, so used a dry heat sterilizer instead, and another area had no functioning sterilisers. A lack of funds for cleaning brushes, detergents, buckets, and protective coverings for instrument storage were also noted.

### Hospital SP assessments

#### Hospital A

Hospital A had 17 ORs from which instruments were transported to a central SP area. There were also several peripheral SP locations, for example in maternity and emergency areas, where the autoclaves reportedly frequently broke, requiring instruments to be transported to other areas for sterilization or sterilized using a dry heat sterilizer. The maternity and emergency units had a small tabletop/countertop autoclave which was used to sterilize small instruments. Workers in the central SP area had received prior SP training, but those working in the peripheral areas (maternity and emergency) had no formal training.

Prior to training, hospital A immersed instruments in bleach. After training the hospital assessment showed that bleach had been replaced by hexanios, an appropriate decontaminating detergent, to remove gross bioburden from instruments.

There was evidence of usage of brushes provided by SPECT, and participant A6 stated that after brushing, instruments “look better, cleaner, and safer to use”. Where prior to training only one sink and bucket had been used to decontaminate, post-training assessments showed they had added a bucket, as they were taught, to create a three-bucket system.

#### Hospital B

Hospital B was a maternity hospital with three ORs. Many of the recommended standards were evident at Hospital B prior to training. Examples include, PPE was worn by staff, 2 autoclaves were working, and surgical site infections were identified and tracked. Prolonged use of bleach, however, was common practice and autoclave guidelines were not followed. Following training the time instruments were soaked in bleach solution was decreased and the autoclave was used according to current guidelines. Participants reported paying closer attention to how they cleaned, placed, wrapped, and stored instruments.

#### Hospital C

Hospital C was poorly resourced with four ORs in various areas of the hospital. Workers in the OR and maternity had no prior training in SP. The hospital did not have a functioning autoclave and relied on dry heat sterilizers. Instruments were washed in buckets on the floor, rinsed with tap water, and left on the floor to dry. Instruments were then placed in a dry heat sterilizer for an inadequate duration at an incorrect temperature. Following training, buckets were placed on a table to process the instruments. The 3-bucket set up and use of brushes to remove bioburden had been implemented and dry heat steriliser times and temperatures were now correct. Bleach was still used due to lack of an appropriate detergent but the soaking time for instruments was reduced to 10 min.

Despite these changes and an increased awareness of good practice, old blood and rust was still found on cleaned and packaged instruments. Also, sterilized instruments continued to be stored in hallways by open windows, increasing the risks of contamination pre-operatively. Further mentoring and support was provided by CF and SV following data collection.

### Knowledge test results

Thirty-three participants completed both the pre-course and post-course knowledge tests. The mean pre-course test score was 57% and post course was 71% (*p* < 0.001). Although the numbers are small, sub-group analysis of the mentored and non-mentored participants showed no significant difference in knowledge gained (mean differences of − 19.2 and − 11.3) between the mentored and non-mentored cohorts. Further details are given in Tables 2 and 3.

**Table 2:** Samples Statistics

	Sample Size	Mean – pre-test	Std Dev	Mean – post-test	Std Dev	correlation	t-value	Sig (1-tail) *p*-value	df	Sig (2-tail)	Range – post-test
Paired Samples	33	57.60%	20.324	71.11%	19.037	0.637	− 4.610	0.119	32	0.000	13–100
Mentored Participants	9	51.85%	20.488	71.11%	16.667	0.705	−3.931	−19.259	8	0.004	33–87
Non-mentored Participants	24	59.72%	20.286	71.11%	20.112	0.628	−3.204	−11.389	23	0.004	13–100

**Table 3:** Paired Samples Test

		Paired Differences					t-value	df	Sig. (2-tailed) (*p*)
		Mean	Std. Deviation	Std. Error Mean	95% Confidence Interval of the Difference				
					Lower	Upper			
Pair 1	Combined Pre – Combined Post-Test	−13.48485	16.80238	2.92492	−19.44271	−7.52698	−4.610	32	.000

## Discussion

Our study shows that a training program in SP changed participants perception of SP and resulted in improved practice at 4 months. These findings provide evidence that even though resources are limited, SP practice can be improved, and SP workers feel more satisfied and valued in their contribution to patient care following SP education.

The WHO standards [1] require SP workers to decontaminate, clean, inspect, package, sterilize, and store instruments in specific ways, paying attention to details that are seldom taught in LMICs. All hospitals in the study were following the WHO [1, 11] global guidelines more closely following education. Participants demonstrated an increased knowledge and sustained improvements in practice 4 months after training. Use of minimal low-cost resources, such as brushes, three buckets and an appropriate detergent, allowed bioburden to be removed from instruments, resulting in decreased risk of patient contamination.

We found no other evidence in the literature on the impact of short SP training courses on SP worker knowledge, job satisfaction and changes in practice. Therefore, comparisons of our data to other training courses are not possible.

A concept that was often lacking prior to the training was that if instruments are not properly cleaned they cannot be sterilized. Cleaning instruments eliminates most bioburden, a process essential for sterilisation. Participants were shocked to learn that when they did not clean the instrument properly, no matter how effective the autoclave was, it would not produce a sterilized instrument, as microbes are able to survive sterilization by hiding under a hardened coat of blood. All participants indicated that not only did they change how they thought about their work, but how they did their work, paying attention to the details in ways not done previously.

As well as improving SP practice, our study shows that training increased participants’ awareness of how SP impacts patient outcomes and also increased participants’ job satisfaction. We did not explore why job satisfaction increased, yet surmise that SP is an essential aspect of patient care, and therefore increased knowledge in SP improved workers’ self-esteem and positive feelings about their work.

A major identified change in practice was decreased exposure of instruments to bleach. Use of bleach to soak instruments immediately after use damages the instruments, is thought to increase resistance to disinfectants, and makes removal of blood and body tissues more difficult [11]. Use of the 3-bucket system to remove bioburden and proper drying and storage of instruments was also an identified change. These are all low-cost initiatives which can easily be implemented as shown in our study.

Our study has some limitations. We report on only three hospitals in one city in Benin therefore transferability and generalizability of findings to other countries may be limited. The short time-scale of follow up and limited sample size of mentored versus non-mentored groups are other limitations. Also, due to the multiple methods used to obtain data it is difficult to identify a specific aspect of the training, either theoretical component or hospital mentoring, that resulted in changes to SP processes and attitudes.

Despite the limitations, our study has a number of strengths. We have provided evidence and unique insight into the impact of a SP training course on knowledge, attitudes, and processes. There is an increasing awareness of the need to address the gap in SP standards between LMICs and high-income countries. O’Hara et al. [2] calls on the international community to fulfill their moral obligation to address barriers to implementing SP guidelines in LMICs. This study addresses that “moral obligation” by providing education and training to those working in SP in LMICs, in a time-bound manner (which may be suitable for wide-spread scale-up) and provides evidence of the effectiveness of this approach. Health care communities must begin to understand that no matter how well a surgery can be performed (including adequate timing of antibiotic administration), if the lack of knowledge and resources related to cleaning and sterilization of instruments is not addressed surgical site infections will continue to be greater in LMICs than high income countries.

## Conclusion

We have shown the positive impact a SP training program has on SP workers and demonstrated sustainable practice improvements at 4 months post-training. We recommend that others involved in safe surgery initiatives, as part of the Global Surgery 2030 agenda [12], pay attention to sterile processing practices in efforts to improve surgical outcomes.

## Additional file

Additional file 1: Infection Prevention Test. (DOCX 68 kb)

## Abbreviations

LMIC: Low Middle-Income Countries
OR: Operating Room
PPE: Personal Protective Equipment
SP: Sterile Processing
SPECT: Sterile Processing Education Charitable Trust
WHO: World Health Organization
